# Drugstore or discount? A study on fluoride, pH and acidity of commercial mouthwashes in Riyadh, Saudi Arabia

**DOI:** 10.3389/froh.2026.1712496

**Published:** 2026-03-30

**Authors:** Rafif Alshenaiber

**Affiliations:** Prosthetic Dental Sciences Department, College of Dentistry, Prince Sattam bin Abdulaziz University, Alkharj, Saudi Arabia

**Keywords:** erosion, fluoride, mouthwash, pH, titratable acidity

## Abstract

**Purpose:**

Evaluation of the fluoride content, pH, and titratable acidity in mouthwashes that are commercially available in Riyadh market from different vendors.

**Method:**

Twenty mouthwashes were randomly collected from different vendors in Riyadh, Saudi Arabia. The fluoride was measured using a fluoride-ion-selective electrode compared with the fluoride content printed on the label. The pH was measured using a digital pH meter, and the titratable acidity was measured by adding 0.1 M increments of sodium hydroxide to attain a pH equal to or greater than the neutral pH. Data were analysed using SPSS statistical software.

**Results:**

There was no significant difference in the fluoride measured between the brands (*p* = 0.445). A significant difference was found between the labeled values and the measured fluoride of all brands (*p*-value = 0.002), with the measured fluoride being less than the labeled values. 6 out of 10 drugstore mouthwashes and 8 out of 10 bargain store mouthwashes showed a pH below 5.5, which is considered potentially erosive. Bargain stores’ mouthwashes showed higher titratable acidity values compared to the drugstore-bought mouthwashes. A significant difference was found between all the mouthwashes’ pH (*p* < 0.001) and titratable acidity (*p* < 0.001).

**Conclusion:**

Some mouthwashes, which are commercially available in the Riyadh market from drugstores and bargain stores, contain fluoride at concentrations lower than the manufacturers’ label value and demonstrate a low pH below the critical threshold for enamel dissolution. Moreover, some of the mouthwashes have high titratable acidity, which poses a potential risk of dental erosion.

## Introduction

Oral health represents an essential component of well-being and significantly impacts the quality of life across the globe (1, 2). The primary action to optimise dental health is to remove the dental plaque biofilm mechanically and chemically. This dental biofilm is considered the primary etiological factor for caries and periodontal disease onset (3). The prophylactic strategy for maintaining dental and periodontal hygiene is a comprehensive routine of limiting dental plaque growth by cleaning the oral cavity using dental brush, toothpaste, inter-dental cleaning aids and mouthwashes.

In Saudi Arabia, a large number of dental and oral care products are available in the market. Such products, including mouthwash, are sold in pharmacies, supermarkets, beauty shops, home goods stores, and bargain stores. A brief inspection of such retail establishments indicates the presence of numerous mouthwash products designed to provide various advantages for oral health. Bargain stores in Saudi Arabia are widely present in each city, which sell goods at lower prices. Such prices are due to the lower operating costs of these establishments, bulk and wholesale purchasing or selling budget-friendly or inferior brands.

Mouthwashes are mostly available without a prescription; some brands are relatively low-priced, while others are expensive. They offer antimicrobial properties with their anticariogenic (4), antiplaque (5) and antihalitosis (6) efficacies. The main therapeutic content of the mouthwash is the active ingredients such as essential oils, cetylpyridinium chloride, chlorohexidine and fluoride (7). However, mouthwashes appear in various colours, flavours, and claims, making it difficult for a common person to understand their efficiency and safety of use. Some researchers found significant discrepancies between the measured content concentration of active ingredients and those specified on the mouthwash label (8–10). Not only the active ingredients but also pH, titratable acidity and other chemical properties of mouthwashes play a key role in their usability and performance in maintaining oral hygiene and are not usually reported on the label. Fluoride is one of the active ingredients of dental care products, and undoubtedly possesses cariostatic and dental tissue remineralizing properties. However, one study conducted in Saudi Arabia found that the real fluoride concentrations were significantly lower than those mentioned on some mouthwash labels (8).

The effects of pH and titratable acidity on dental health compared to microbial activity are inspected. The literature has frequently reported that slightly acidic mouthwashes are effective against oral bacteria (4–6, 11). In view of this, it is necessary to understand the pH and the titratable acidity well to evaluate the potential erosion of mouthwash and the proper use of consumer oral care products. Understanding the importance of titratable acidity testing is associated with the recognition of the buffering capacity of saliva. Therefore, mouthwashes exhibiting low titratable acidity are easily neutralised by saliva, whereas those possessing high titratable acidity result in a sustained decrease in oral pH, potentially resulting in the demineralisation of dental tissues (12, 13).

Local and international companies manufacture the mouthwashes available in the Saudi market. However, it is obligatory to report the list of mouthwash ingredients in Arabic on the package as per The Saudi Food and Drug Authority (SFDA). To regulate the mouthwash manufactured and sold in Saudi, they adopted the ISO 16408:2015 standards for dentistry, oral care products, and oral rinses (14). In addition, the drug sector of SFDA has banned the manufacturing or importing of alcohol-containing mouthwashes to Saudi markets. The ISO 16408:2015 standards specify that the pH value of the mouthwash should be tested at its intended concentration to be between 3 and 10.5. Moreover, if the pH is less than 5.5, it should be tested further using ISO 28888 standards for dentistry—screening method for erosion potential of oral rinse on dental hard tissues (15).

Mouthwashes are commonly used along with brushing and flossing the teeth during oral care routine. In a study of 1,259 participants in Saudi to assess the prevalence of mouthwash use, 83% reported that they had used mouthwash, and 34% reported that their dentist advised them to use it, while more than 40% used mouthwash due to self-awareness (16). Nevertheless, high caries and periodontal disease are reported among Saudi adults and children (17–19). Unfortunately, 40% of Saudis stated that they do not brush their teeth, and 16% never used interdental aids (20). Likewise, using mouthwash is not common, as 17% of Saudis said they have never used mouthwash, and only 11% of people use it as a habit (16). On the other hand, the vast, excessive and incorrect use of mouthwashes by the general population created a significant concern due to the high acidity of some mouthwash products, which potentially causes dental erosion (21, 22). Moreover, using some over-the-counter mouthwashes may result in an imbalance within the oral microflora (23).

In the dental literature, several studies have focused on evaluating the contents and the physicochemical properties, such as pH and titratable acidity of mouthwashes available in the international market (12, 24, 25). The comprehensive study about the fluoride content and the characteristics of pH and titratable acidity of the available mouthwashes is not publicly known to Saudi consumers. To the best of the authors’ knowledge, there is no scientific study about fluoride content, pH and titratable acidity of mouthwashes from different Saudi vendors. This study aims to analyse fluoride concentration and compare it to the label values and to the recommended fluoride concentration in mouthwash, and examine the physicochemical properties of pH and titratable acidity of some mouthwashes obtained from different vendors (drugstores and home bargain stores) in Riyadh, Saudi Arabia.

## Materials and methods

Ethical clearance was obtained from Prince Sattam bin Abdulaziz University Institutional Review Board. Three bottles from twenty distinct commercial mouthwash brands were randomly purchased over the counter (OTC) from different vendors (*n* = 60). Ten brands from drugstores and ten from bargain stores in Riyadh, Saudi Arabia. All mouthwashes studied were collected between October and December 2024 and checked for manufacturing and expiry dates prior to testing. Since purchasing, the mouthwashes were transported in a car and stored at room temperature (20 °C–25 °C) till all samples were collected and the test commenced on all at the same time. The brand, active ingredients, and manufacturer data and fluoride content per the label were reported (Table 1). The bottles commercially available are comprised of various active ingredients, ranging between 250 mL and 300 mL, with a range price of 8.5 Saudi riyal for those bought from drugstores and 2 Saudi riyal from bargain stores for each 50 mL mouthwash.

**Table 1:** List of mouthwashes according to the commercial brand, type, active ingredients, fluoride content, manufacturer and place of purchase.

No.	Commercial brand and type	Active ingredient	Fluoride content per the label	Manufacturer	Place of purchase
1	Listerine, cool mint daily mouthwash	Thymol	Sodium fluoride 220 ppm	Johnson & Johnson	Drugstore
2	Oral-B mouthwash pro expert deep clean	Cetylpyridinium chloride	Sodium fluoride 100 ppm	Procter and gamble	Drugstore
3	Colgate-peroxyl mouth sore rinse	Hydrogen peroxide	None	Colgate -palmolive	Drugstore
4	Lacalut® active	Chlorhexidine digluconate	Sodium fluoride 225 ppm	Lacalut	Drugstore
5	Meridol® gum protection	Amine fluoride	Sodium fluoride 254 ppm	Colgate-palmolive	Drugstore
6	EZ fresh	Chlorhexidine diacetate	None	Avalon pharma	Drugstore
7	AloeDent triple action	Grapefruit seeds extract, melaleuca alternifolia (tea tree) leaf oil	None	AloeDent®	Drugstore
8	Astera daily	Cetylpyridinium chloride	Sodium fluoride 225 ppm	Astera	Drugstore
9	CloseUp—antibacterial mouthwash	Sodium fluoride	Sodium fluoride 225 ppm	CloseUpUnilever	Drugstore
10	Fluorodine active plus	Zinc chloride	Sodium fluoride 225 ppm	Fluorodine	Drugstore
11	Beauty system mouthwash capsules	Cetylpyridinium chloride	None	Beauty system	Bargain stores
12	Gargaline	Thymol	None	Gargaline	Bargain stores
13	Amalfi classic	Chlorhexidine digluconate	Sodium fluoride 220 ppm	Quimi Romar S.L	Bargain stores
14	President® classic	Chlorhexidine digluconate	Sodium fluoride 230 ppm	BETAFARMA Spa	Bargain stores
15	Parodont active	Chlorhexidine gluconate	Sodium fluoride, 450 ppm	Parodont	Bargain stores
16	Eludril care	Cetylpyridinium chloride	None	Pierre Fabre	Bargain stores
17	Oradent fresh burst	Thymol	None	Leader Pharm	Bargain stores
18	Pierrot sensitive	Cetylpyridinium chloride	Sodium fluoride 225 ppm	Pierrot	Bargain stores
19	Oroguard	Chlorohexamine gluconate	Sodium fluoride 225 ppm	Alkem laboratories Ltd	Bargain stores
20	Oralgin	Chlorhexidine gluconate	Sodium fluoride 225 ppm	Oralgin	Bargain stores

Before the testing, all three mouthwash bottles from the same brand were fully concealed inside one numbered large box from 1 to 20 by a research assistant to allow blind analysis by a single main researcher (RA) who carried out all the testing. The measurements of the total soluble fluoride, pH, and titratable acidity were tested at room temperature (20 °C–25 °C) and carried out using the means of the measurements from the three different bottles of each mouthwash brand.

Prior to the measurement of fluoride concentration, a fluoride-selective electrode (HI4110, Hanna Instruments, Woonsocket, USA) was calibrated using the same volume ratio of 1:1 made from 1 mL of the total ionic strength adjustment buffer solution (HI4010-00, TISAB II for Fluoride ISE, Hanna Instruments, Woonsocket, USA) mixed with 1 mL of fluoride standards (HI4110 Fluoride ISE 0.1M Standard 0.1M, Hanna Instruments, Woonsocket, USA) for two minutes using (Hanna HI 180 magnetic stirrer, Hanna Instruments, Woonsocket, USA). This fluoride-selective electrode is suitable for measuring small volume samples and connected to a laboratory meter (Hanna HI 4222 pH/ISE/mV meter, Hanna Instruments, Woonsocket, USA), which measures the pH at the same time. The Hanna HI 4222 pH/ISE/mV meter was also calibrated using pH calibration aqueous buffer solutions (Thermo Scientific™ Orion™, USA) at pH 7, pH 4.01 and pH 10.01, respectively.

The total soluble fluoride was then measured using the same method, mixing 5 mL of HI4010-00, TISAB II solution with 5 mL of each mouthwash for two minutes, then immersing the fluoride-selective electrode in the mixture for 10 min to allow for the stabilisation of the recorded value. The F values plotted on semi-log paper from the Hanna HI 4,222 meter in millivolts (mV) were converted to concentration in ppm (mg/L) from the plot. Three measurements from the three bottles were taken for all mouthwash brands for a total of 9 readings for the fluoride and 9 readings for pH. The ion-selective electrode was rinsed using deionised water of pH 7.5 and patted dry between each measurement to prevent carryover or sample contamination.

The Hanna HI 4,222 meter was also used to measure the titratable acidity by titrating 25 mL of the mouthwash with 0.1 M sodium hydroxide (NaOH) solution at a rate of 1 mL per minute until close to pH 6, then 0.05 mL every 10 s to attain a pH equal to or greater than the neutral pH of 7. The measurement was repeated three times on three bottles, and the mean was calculated. If the pH of the mouthwash was 7 or greater when measured initially, the titratable acidity was not recorded.

Using IBM SPSS statistical software 25 (SPSS Inc., Chicago, IL, USA), the data distribution was assessed using Shapiro–Wilk test. The mean and standard deviations of fluoride concentration, pH, and titratable acidity from 9 measurements for each brand were statistically analysed. The statistical significance of fluoride concentration, pH, and titratable acidity between the brands was measured using the Kruskal–Wallis test (*p* < 0.05) and *post hoc* tests in case of significance. The Wilcoxon signed-rank test was used to analyse the following: the difference between the measured fluoride concentration and the fluoride concentration on labels of each mouthwash containing fluoride (*p* < 0.05), the difference between the measured fluoride concentration and the fluoride concentration of 230 ppm recommended for daily use in a mouthwash (*p* < 0.05) (26). Wilcoxon signed-rank was also used to analyse the difference between the measured pH and titratable acidity, with the critical value of 5.5 pH (*p* < 0.05).

## Results

The data on total soluble fluoride, pH, and titratable acidity values were non-normally distributed. Table 2 shows the mean values and standard deviations of the measured total soluble fluoride, pH, and the titratable acidity of the mouthwashes. It also shows the presence of fluoride, type, and concentration per the label.

**Table 2:** The fluoride content, mean ± standard deviation (SD) of the pH and titratable acidity of the tested mouthwashes.

No.	Commercial brand and type	Fluoride content measured mean ± SD	pH mean ± SD	Titratable acidity mean ± SD
1	Listerine, cool mint daily mouthwash	210.06 ± 1.70	4.52 ± 0.12	7.07 ± 0.01
2	Oral-B mouthwash pro expert deep clean	169.09 ± 2.11	4.79 ± 0.22	0.45 ± 0.01
3	Colgate-peroxyl mouth sore rinse	–	3.56 ± 0.06	0.05 ± 0.01
4	Lacalut® active	204.19 ± 4.20	5.82 ± 0.07	0.21 ± 0.01
5	Meridol® gum protection	216.03 ± 2.01	4.31 ± 0.03	5.75 ± 0.15
6	EZ fresh	–	4.62 ± 0.23	0.9 ± 0.10
7	AloeDent triple action	–	6.71 ± 0.10	0.7 ± 0.10
8	Astera daily	209.52 ± 1.07	5.70 ± 0.03	0.2 ± 0.10
9	CloseUp—antibacterial mouthwash	210.08 ± 2.02	6.87 ± 0.01	0.2 ± 0.00
10	Fluorodine active plus	202.06 ± 3.10	4.33 ± 0.31	0.03 ± 0.01
11	Beauty system mouthwash capsules	–	3.40 ± 0.02	5.38 ± 0.03
12	Gargaline	–	4.36 ± 0.09	3.09 ± 0.10
13	Amalfi classic	181.00 ± 3.98	4.62 ± 0.34	2.06 ± 0.00
14	President® classic	212.09 ± 4.02	5.10 ± 0.03	0.07 ± 0.01
15	Parodont active	411.31 ± 2.52	4.48 ± 0.33	0.25 ± 0.01
16	Eludril care	–	6.80 ± 0.33	0.20 ± 0.01
17	Oradent fresh burst	–	4.56 ± 0.23	1.02 ± 0.01
18	Pierrot sensitive	207.08 ± 2.04	5.79 ± 0.03	4.53 ± 0.03
19	Oroguard	208.03 ± 1.06	3.87 ± 0.29	5.02 ± 0.00
20	Oralgin	219.01 ± 2.01	4.36 ± 0.09	4.07 ± 0.03

Seven brand labels showed no presence of fluoride in the contents. The remaining 13 mouthwash labels indicated the presence of fluoride and its concentration. The fluoride concentrations from the labels ranged from 100 ppm (Oral-B Mouthwash Pro Expert Deep Clean) to 450 ppm (Parodont Active). In contrast, the fluoride concentrations measured in this study ranged from 169.09 ppm (Oral-B Mouthwash Pro Expert Deep Clean to 432.31 ppm (Parodont Active).

Kruskal–Wallis test indicated no significant difference in the fluoride concentrations measured between the brands (*p* = 0.445). However, the Wilcoxon signed-rank indicated a significant difference between the labeled values and the measured fluoride concentrations of all brands (*p*-value = 0.002). All brands’ fluoride concentrations measured were significantly lower than the label value, except for Oral-B Mouthwash Pro Expert Deep Clean, which exceeded the labeled value of 100 ppm fluoride, with a measured value was 169.09 ppm. There was no significant difference between the measured fluoride of all brands and the recommended concentration in daily mouthwashes of 230 ppm.

A significant difference using Kruskal–Wallis test was found between all the mouthwashes’ pH and titratable acidity, *p* < 0.001 and *p* < 0.001, respectively. *post hoc* comparisons of the mouthwashes’ pH showed that (Close-up) and (Beauty System) are significantly different from other brands. The titratable acidity *post hoc* comparisons indicated that (Listerine) and (Colgate-Peroxyl) are significantly different from other brands. Wilcoxon signed-rank test showed that among the 20 mouthwashes evaluated, only 6 (Lacalut, AloeDent, Astera, CloseUp, Eludril, Pierrot) had a significantly higher pH value compared to the critical value of 5.5. Therefore, the other 14 mouthwashes can be considered potentially erosive as they scored a pH of less than 5.5.

Based on the vendors, (4/10) of drugstores bought mouthwashes showed significantly higher pH values above 5.5, while (2/10) of bargain stores bought mouthwashes showed a pH above 5.5. For the titratable acidity, only two mouthwashes (Listerine and Meridol) exhibited significantly higher titratable acidity values than the drugstore-bought mouthwashes. In comparison, bargain stores bought mouthwashes, 7 mouthwashes, including Beauty System, Gargaline, Amalfi, Oradent, Pierrot, Oroguard, and Oralgin all of which exhibited significantly higher titratable acidity values.

## Discussion

To some people, a typical daily oral health routine includes brushing and flossing, often supplemented with the use of mouthwash. Mouthwash can be used as it is or diluted in water according to the manufacturer's instructions (27). Some types of mouthwash are used only before brushing teeth, while others can be used after (12). Fluoride mouthwash is recommended for some patients with a high risk of caries. The recommended concentration of fluoride in these mouthwashes used daily is 230 ppm to prevent caries (26). The measured fluoride of all brands from both vendors showed no significant difference from the recommended concentration in daily mouthwashes, indicating that all prevent caries. In this study, there was a significant difference between the labeled values and the measured fluoride concentrations of all brands except one, which was bought from a drugstore, implying there was less fluoride available than labeled. The difference in concentration can be due to the different fluoride types. One study found that Sodium Fluoride (NaF) dissolves rapidly in water compared to monofluorophosphate (MFP) due to the bond between the fluoride ion and the phosphate group in MFP, which prevents the release of fluoride ion from being measured without further treatment of the solution (28). However, the fluoride content in this study of all brands was NaF per the manufacturers, hence the discrepancy in the measured fluoride results and labeled is not related to the fluoride formulation, but it indicates lower fluoride content than marketed.

Although considered scientifically advantageous to the teeth (29), no fluoride mouthwashes are available and were analysed in this study. Many have shown their opposition to fluoride due to its toxicity (30). Several countries have changed their policies towards water fluoridation due to professional and public demands (31, 32). Therefore, future studies should involve the analysis of no-fluoride mouthwashes vs. fluoride-containing mouthwashes, particularly with relation to reduced pH. In the literature, several studies showed that some types of mouthwashes are acidic (12, 24, 25). Frequent and prolonged exposure to acidic mouthwashes may result in the dissolution of dental tissues (12, 13). Mouthwashes with a pH of 5.5 or less were considered potentially erosive (24, 33, 34). Therefore, the use of mouthwash with low pH should be avoided before brushing to reduce erosion of dental tissues, which can be worsened with mechanical brushing. The effect is not solely on dental tissue, but fluctuation in the pH level in the mouth can cause alterations in normal oral flora and affect the ecological equilibrium (23). Moreover, one study found that the consistent use of OTC mouthwash was correlated with the risk of developing pre-diabetes or diabetes in patients (35). Regular use of mouthwash was also associated with an increased risk of oral cancer (36). Therefore, the daily usage of some mouthwashes may lead to harmful results. Although potentially harmful to dental tissues, an acidic pH favours the antibacterial properties of the mouthwash (4–6, 11) and increases the chemical stability of fluoride in the mouthwash (37). Therefore, when combined with low pH, the lack of fluoride in the mouthwash ingredients suggests that such mouthwash cannot be used for the long term. In this study, two out of three drugstore mouthwashes showed low pH and lack of fluoride (Colgate and EZ Fresh), while three out of four bargain store mouthwashes showed low pH and lack of fluoride **(**Beauty System, Gargaline, and Oradent).

Determining pH is one of the most important and commonly performed analyses of dental care products. Although it is a relatively simple technique, every precaution must be taken to obtain reliable and accurate results. However, the determination of the titratable acidity is the most significant indicator of the potential for enamel erosion. In this regard, the term acidity is more accurately aligned with titratable acidity when discussing pH. Among the tested mouthwashes in this study, there was considerable variation in pH and the titratable acidity values. Fourteen mouthwashes scored a pH below the critical pH of 5.5; hence, they were considered potentially erosive (24, 33, 34). The titratable acidity is related to saliva's buffering ability to neutralise the mouth's pH. Most of the bargain stores’ mouthwashes exhibited high titratable acidity values, causing a prolonged drop in the pH. Subsequently, the acidic pH will last longer in the mouth, posing a greater risk of dental erosion. Therefore, caution should be taken when patients with xerostomia or consume an acidic diet and use mouthwashes with low pH or high titratable acidity. Such results may have implications for oral health efficacy and safety guidelines for consumers.

Considering consumer behaviour, some people possess awareness, and some do not pay attention to what they are using for oral care. A study that was carried out in different developed and developing countries, including Saudi Arabia, found an increasing interest among online users in searching for mouthwash-related topics between 2004 and 2020 (38). However, more than half of mouthwash users in Saudi Arabia do not care about using a specific mouthwash brand (16). Such a result may indicate that consumers lack knowledge regarding what mouthwash they should use or consider other factors in their choice. In this study, all bargain stores’ mouthwashes were cheaper than those bought from drugstores. In a questionnaire conducted in Canada, about 39% reported that the price of mouthwash affected their decision to buy it (39).

All mouthwashes available on the Saudi market provide their contents per SFDA regulation. Hence, all mouthwashes containing fluoride were labelled with the fluoride content and concentration. On the other hand, the pH and titratable acidity values are not mentioned on the mouthwash labels. However, the hazardous use of some mouthwashes due to the high acidity should impel the health authorities in each country to implement regulations to declare the pH and titratable acidity on the label. Moreover, collaborations between industry, academia, and the health sector are necessary for increasing the customer's awareness of the importance of choosing the most proper mouthwash and usage based on informed knowledge.

The American Dental Association (ADA) describes mouthwash as a cosmetic and therapeutic product (40). Both types are commercially available in the Saudi market and can be purchased from several vendors. Non-pharmaceutical retail vendors in Saudi Arabia sell OTC oral health care products, including mouthwashes. However, such vendors may lack the proper storage conditions and proper personnel to guide usage compared to pharmaceutical retail vendors. One study in the United Kingdom showed a deficiency in the regulation of selling OTC health products in non-pharmaceutical retail vendors (41).

Different types of mouthwashes are intended to be used differently; some are anti-caries, while others are used to prevent periodontal diseases. One limitation of this study is the lack of sub-classification of mouthwash brands based on their intended use. The outcomes of this study cannot be directly applied to clinical scenarios, as several factors, such as the quality and quantity of the oral fluid, saliva consistency and pH, patients’ habits, and timing of mouthwash use, were not considered. Moreover, the buffering capacity of patients’ saliva was not considered, and further *in vitro/vivo* studies are needed to verify the results, particularly regarding the critical pH of 5.5.

This study provided a base for an intensive survey of content and formulations, especially pH and titratable acidity of mouthwashes that are present in the Saudi market, to provide a database for both professionals and consumers. One international review reported that pharmacists and pharmacy staff lack knowledge, education and training about dental and oral health care (42). In Saudi Arabia, only 12.5% of pharmacists practising in Riyadh know that dental plaque is a soft deposit on teeth (43). Furthermore, there is a need for research to investigate the potential long-term effects of regular mouthwash usage based on the pH and titratable acidity.

## Conclusion

Some mouthwashes, which are commercially available in Riyadh market, Saudi Arabia, from drugstores and bargain stores, have demonstrated a low pH below the critical threshold for dental erosion. Some have no fluoride, which may suggest that such mouthwash cannot be used for the long term, and those containing fluoride contain less fluoride than labeled, but all contain the recommended concentration of fluoride in daily mouthwashes. Additionally, they exhibited a high titratable acidity, which poses a potential risk of dental erosion if not utilised appropriately.

## Data Availability

The original contributions presented in the study are included in the article/Supplementary Material, further inquiries can be directed to the corresponding author.
